# Developing a feasible and valid scoring system for critically ill patients in resource-limited settings

**DOI:** 10.1186/s13054-017-1902-z

**Published:** 2018-01-05

**Authors:** Gentle Sunder Shrestha

**Affiliations:** Department of Anaesthesiology, Critical Care Unit, Tribhuvan University Teaching Hospital, Maharajgunj, Kathmandu Nepal

In the study by Haniffa et al., the authors have brilliantly proposed “TropICS” as the first multinational prognostic model for critically ill patients in resource-limited settings. The study used data from four South East Asian nations, involving a large number of unselected ICU patients for development and validation of three prognostic models. Of the three proposed models, TropICS performed better than APACHE II and SAPS II in terms of discrimination and calibration. It is interesting to see them test the three models, which varied in inclusion of clinical, laboratory and treatment variables, which were selected based on multivariate logistic regression. The models have tried to address the variable availability and affordability of the parameters measured [1].

However, it would be prudent to note that the complete case availability for APACHE II was only 15% [1]. Moreover, as diagnostic, therapeutic, and prognostic techniques in the ICU evolve over time, the scoring systems need to be updated [2]. Newer APACHE versions, such as APACHE IV, which is based on a larger database and in which the selection of variables and their weights is based on multiple logistic regression, perform better than older versions [3]. These factors together might have caused the lower performance of APACHE II compared to the other proposed models.

Disease-specific scoring systems are increasingly being used. The SOFA score has been proposed to quantify organ dysfunction, as per Sepsis-3 [4]. Some of the parameters of the SOFA score like PaO_2_, serum creatinine, and bilirubin level have poor availability (less than 50%) in the databases of the new models, raising questions about the feasibility and validity of using the SOFA score to quantify organ dysfunction in resource-poor settings [1]. There may be a need to develop a simpler and more feasible scoring system to recognize sepsis in resource-limited settings [5]. Laboratory variables of TropICS, blood urea and hemoglobin level, were available for only 50% of the patients [1]. Considering the wide variability of availability of resources between low and middle-income countries (LMICs) and even within the same nation, TropICS needs to be validated across these settings before assuming global applicability in places with limited resources.

Rather than depending on a single score at admission to ICU, change in score over time (like the delta SOFA score) may reflect the progression of organ dysfunction over time and can be helpful for better prognostication [2]. Future studies may attempt to explore the utility of the delta score for TropICS as well.

## Abbreviations

APACHE: Acute Physiology and Chronic Health Evaluation
ICU: Intensive Care Unit
SAPS: Simplified Acute Physiology Score
SOFA: Sequential Organ Failure Assessment.
TropICS: Tropical Intensive Care Score
