# Supplementary material for: Developing a feasible and valid scoring system for critically ill patients in resource-limited settings
Source: Crit Care. 2018 Jan 5;22:2. doi: 10.1186/s13054-017-1902-z (PMC5756425; doi:10.1186/s13054-017-1902-z)
Supplement: Related texts

# Reply: Authors’ response

**Authors:** Rashan Haniffa, Mavuto Mukaka, Sithum Bandara Munasinghe, Ambepitiyawaduge Pubudu De Silva, Kosala Saroj Amarasiri Jayasinghe, Abi Beane, Nicolette de Keizer, Arjen M Dondorp

We would like to thank Dr. Shrestha for his insightful comments. Our systematic review attributes the variable performance of prognostic models in LMICs to three main factors: missing data and subsequent normal imputation; differing endpoints due to unknown hospital outcomes; and the inadvertent inclusion of paediatric patients [6]. Other reasons more commonly attributed to poor performance of prognostic models in such settings include differing case-mix when compared to high income countries (HICs) and a predominantly healthy and younger population. These are, however, often difficult to evaluate given the limited investment in infrastructure (for example medical registries) enabling the systematic collection of data. We agree that the reason for the poor performance of APACHE II in our dataset may be due to missing data [7]. However, for the same reason, newer and more complicated versions such as APACHE III or IV are unlikely to fare any better given their greater data collection burden. It is also important to note that such missingness is not limited to LMICs or indeed to the ICU [8]. The development of simplified models such as TropICS, R-MPM and qSOFA can be seen as attempts to overcome this impediment.

As variability between and within countries is likely to persist, and as all models require regular validation and refinement (for example by recalibration), prognostic model selection and assessment are perhaps better guided, at least initially, by data availability; by matching models and their variants to the degree of missingness [7]. Subsequent validation using the classic statistical tests of discrimination, calibration and accuracy can then follow. Further research to investigate the impact of the extent of missingness on prognostic models and strategies to mitigate this seemingly ubiquitous problem, beyond traditional statistical methods, is ongoing in multiple LMIC settings by our group.

In addition to the quest to refine statistical methodology to improve model performance, we recommend that the validation of such tools should be seen in the context of ‘real world performance and impact on patient outcomes’ [9]. To have greater applicability to improving the quality and equity of care provided, the priorities of clinicians, researchers and administrators in the target setting need to be better understood. Could the biggest barrier to the validity and applicability of such tools be the disconnect of the necessary stakeholders from processes established as indispensable in HICs: benchmarking, audit and quality improvement, and risk stratification for clinical trials?
